# Anti-*Trypanosoma cruzi* Cross-Reactive Antibodies Detected at High Rate in Non-Exposed Individuals Living in Non-Endemic Regions: Seroprevalence and Association to Other Viral Serologies

**DOI:** 10.1371/journal.pone.0074493

**Published:** 2013-09-17

**Authors:** Esber S. Saba, Lucie Gueyffier, Marie-Laure Dichtel-Danjoy, Bruno Pozzetto, Thomas Bourlet, François Gueyffier, Yahia Mekki, Hans Pottel, Ester C. Sabino, Philippe Vanhems, Maan A. Zrein

**Affiliations:** 1 InfYnity-Biomarkers, Ecully, France; 2 Laboratory of Bacteriology-Virology, GIMAP EA3064, Faculty of Medicine Jacques Lisfranc, Saint-Etienne, France; 3 UCBL-Hospices Civils de Lyon, Faculty of Medicine Rockefeller, Lyon, France; 4 Interdisciplinary Research Center, Catholic University Leuven, Campus Kortrijk, Kortrijk, Belgium; 5 Faculdade de Medicina da USP, Dep de Molestias Infecciosas e Parasitárias, São Paulo, Brazil; INSERM U1094, University of Limoges School of Medicine, France

## Abstract

Cross-reactive antibodies are characterized by their recognition of antigens that are different from the trigger immunogen. This happens when the similarity between two different antigenic determinants becomes adequate enough to enable a specific binding with such cross-reactive antibodies. In the present manuscript, we report the presence, at an “abnormal” high frequency, of antibodies in blood samples from French human subjects cross-reacting with a synthetic-peptide antigen derived from a *Trypanosoma cruzi* (*T. cruzi*) protein sequence. As the vector of *T. cruzi* is virtually confined to South America, the parasite is unlikely to be the trigger immunogen of the cross-reactive antibodies detected in France. At present, the cross-reactive antibodies are measured by using an in-house ELISA method that employs the *T. cruzi* -peptide antigen. However, to underline their cross-reactive characteristics, we called these antibodies “*Trypanosoma cruzi* Cross Reactive Antibodies” or TcCRA. To validate their cross-reactive nature, these antibodies were affinity-purified from plasma of healthy blood donor and were then shown to specifically react with the *T. cruzi* parasite by immunofluorescence. Seroprevalence of TcCRA was estimated at 45% in serum samples of French blood donors while the same peptide-antigen reacts with about 96% of *T. cruzi* -infected Brazilian individuals. In addition, we compared the serology of TcCRA to other serologies such as HSV 1/2, EBV, HHV-6, CMV, VZV, adenovirus, parvovirus B19, mumps virus, rubella virus, respiratory syncytial virus, measles and enterovirus. No association was identified to any of the tested viruses. Furthermore, we tested sera from different age groups for TcCRA and found a progressive acquisition starting from early childhood. Our findings show a large seroprevalence of cross-reactive antibodies to a well-defined *T. cruzi* antigen and suggest they are induced by a widely spread immunogen, acquired from childhood. The etiology of TcCRA and their clinical relevance still need to be investigated.

## Introduction

The paradigm of antibody specificity is closely related to the primary amino-acid sequence forming the heavy and light chains in a spatial organization that is able to bind to a given antigenic structure. However, each individual antibody molecule has a built-in capability to bind to various antigenic motifs; this non-specific recognition can gradually attain degeneracy where an antibody molecule is able to bind to fairly distant antigens. Nevertheless, the specificity is accomplished when the sum of specific bindings to a given antigenic determinant is clearly superior to the cross-reactive bindings to a variety of different structures. This is typically obtained in polyclonal antisera.

An important cause of cross-reactivity is attributable to molecular mimicry between antigenic structures. Thus, an infective agent can partially mimic tissue-specific antigens and induce cross-reactive autoimmune antibodies. Antigen mimicry can drive an immune response, initially directed against a foreign antigen, to recognize the host antigens and then results in dysfunction and autoimmune diseases. Such mechanisms have been proposed to explain certain acquired immune pathogenesis [1] [2].

In the context of an infection by *T. cruzi*, either the parasite and/or the associated polyclonal reactivity ultimately lead to Chronic Chagas Cardiomyopathy (CCC) in about 30% of infected people 10 to 30 years after the infection [3]. The detection of *T. cruzi* nests in the heart of patients with chronic myocarditis suggests the persistence of the parasite as a cause for the development of CCC [4] Conversely, other researchers reported unsuccessful parasite detection in a great majority of patients with CCC which constitute a doubt about the necessity of the parasite for the development of Chagas pathology [5]. Furthermore, several reports indicate that the inflammatory tissue damage may not be correlated to the local presence of *T. cruzi* [6] [7]. Evidence for a direct pathogenic role of autoimmunity was suggested by the development of lesions in cardiac tissues after immunization with *T. cruzi* antigens in animal models [8]. Several *T. cruzi* antigens have been reported to present epitopes similar to mammalian antigens, including the family of trypanomastigote specific FI-160 antigens [9], cruzipain [10], calreticulin [11], SAPA [12], members of the ribosomal P protein family, and many other antigens (for a review see [3]). Aside from the controversial pathogenesis that leads to CCC after *T. cruzi* infection, in laboratory diagnostic testing, several cross-reactive antigens have been described to produce false reactivities in Chagas screening serological assays [13]. Some of them were observed to bind with antibodies induced by parasites belonging to the member of the same trypanosomatids group like for Leishmania [14] and also by more distant parasites like Malaria [15]. Cross reactivity is depending on the source of *T. cruzi* antigens used in the immunoassays development (recombinant proteins and synthetic peptides, or crude extracts from *Trypanosoma cruzi* epimastigote forms), however in such assays the frequency of cross-reactivity remains extremely limited due to regulatory considerations.

In the course of development of a new serodiagnostic assay for Chagas Oelemann et *al* observed a strong cross-reactivity of an antigen that we further called TCSP for *Trypanosoma cruzi* Synthetic Peptide [16]. This peptide belongs to the repetitive region of the 60 S L19 ribosomal protein of *T. cruzi* [17]. This repetitive region was initially described in an attempt to determine antigenic sequences of *T. cruzi* [18]. Repeated motifs are found in several members of the 60S ribosomal proteins [19]. The largest C terminal extensions (more than 160 amino acids) have been observed in *T. cruzi* L19 and *T. cruzi* S21 and are specific to trypanosomatids [20]

The objective of the present work is to describe the seroprevalence of cross-reacting antibodies to TCSP in a non-endemic region for *T. cruzi*. These antibodies are unexpectedly found at a high seroprevalence (40% to 50%) in serum of individuals living in France, not exposed to *T. cruzi*. They have thus been named TcCRA for *Trypanosoma-cruzi*-Cross Reactive Antibodies. Cross-reactivity of these TcCRA antibodies to *T. cruzi* is also demonstrated. These initial observational studies may help in further exploring potential association of TcCRA with diseases suspected but not yet proved to have an infectious origin.

## Materials and Methods

### Ethics Statement

The Institutional Review Board we depend upon waived the study approval (CPP Sud-Est n° 2013/017). The sera that were tested indeed represented residual quantities from samples withdrawn for other purposes and all sera were anonymized prior to testing.

All our studies comply with the French legislation on the processing of personal data and have been declared to the competent authority (CNIL – National Commission for Information technology and Liberty).

### *T. cruzi* -synthetic peptide (TCSP) antigen

The peptide sequence of 19 amino-acids is coupled to bovine serum albumin (BSA) and has the following sequence: BSA-AAAPAKAAAAPAKTAAAPV.

The peptide synthesis was performed using Fmoc-chemistry supplied by Protéogenix, France. The peptide was then covalently linked from the N-terminal side to bovine serum albumin (BSA) to facilitate its adsorption to microplates. This peptide is used as a target antigen throughout our work to detect TcCRA.

### BLAST searches and Alignments

We performed a BLAST search using the Universal Protein Resource (www.uniprot.org), by querying first the TCSP peptide, then the repeated region harboring the peptide. This region, referenced as Q7M3W1 (97 amino-acids), is a fragment of *T. cruzi* ribosomal protein 60s L19 (XP_808122; 357 amino-acids). We searched across the Swiss Prot non-redundant database sequences by using the default settings [21].

### Human serum collections

We tested TcCRA presence in 395 serum samples that were routinely collected for viral serology testing of one or more parameter(s) in a hospital setting (Lyon, France). We retained serum samples if a minimum of 0.5 mL was available.

In addition, 210 serum samples from healthy blood donors were obtained from different blood banks (Etablissement Français du Sang (EFS) Rhône, EFS Nord and EFS Auvergne Loire). All samples from blood donors were screened negative for the following infectious serological markers, namely: human immunodeficiency viruses (HIV 1, 2 & O), Human T-lymphotropic viruses (HTLV I/II), Hepatitis B virus (HBV), Hepatitis C virus (HCV) and *Treponema palladium* (TP). Moreover, blood donors are classically asked before sampling whether they have travelled to any of the endemic regions for *T. cruzi*. In case they have, their blood is tested for the presence of the parasite infection and their sample is discarded if found reactive.

Additionally, we tested 69 serums of pediatric patients (< 18 years) obtained from Amiens biobank, as well as 79 sera of *T. cruzi* -infected patients obtained from Fundação ProSangue (São-Paulo, Brazil).

Sex and age at sampling were scored for all samples (Table 1).

**Table 1:** Demographic variables of the tested samples.

Tested samples from	Age, mean years (SD)	Gender, M/F
Hospital sources	37.5 (21.6)	229/166
Blood donors	39.5 (14.7)	134/76
*T. cruzi* infected patients	47.5 (8.5)	45/34
Pediatrics (< 18 years)	9.7 (6.0)	36/33

### Serology testing

Antibodies specific to the TCSP peptide antigen were tested in serum samples using an in-house ELISA method. Briefly, TCSP was coated at a concentration of 1 µg/ml in 100 µL coating buffer pH 9.6 into 96-wells microplates (Nunc-Immuno™). Microplates were blocked with 200 µl of blocking buffer during 2 h at room temperature. Following incubation, the microplates were washed two times with ELISA wash buffer (PBS-Tween) using an automated washer (Labatech LT-3500). Samples were then diluted appropriately in sample diluents to 1/50. In brief, 100 µl of diluted sample or controls were then added to TCSP coated plate and incubated for 60 min at room temperature. At the end of the incubation, the plates were washed three times with wash buffer to remove unbound antibodies (LT-3500 Microplate washer). Alkaline Phosphatase-conjugated (PARIS BIOTECH, Compiègne, France) was diluted 1/1000 in conjugate buffer, and 100μl were then added to the microplate which was incubated for a final 60 min at room temperature. At the end of the incubation period, the plates were washed three times with wash buffer. Finally 100μl of *para*-Nitrophenyl Phosphate (p-NPP) substrate solution (1 mg/mL) were added to each well. The plate was then incubated at room temperature for 15 min. The absorbance was measured at 405 nm using a 96-well plate reader (Labatech LT-4000 Microplate reader) and the results analyzed using analysis software (Manta, Labatech). All reported data represent the average optical density (OD) of duplicate measurements. A cut-off value of 0.5 OD was calculated for determining seropositivity, it is determined as the mean plus 3 times the standard deviations (SD) of the tested samples OD on negative control samples.

Viral serologies for clinical samples were performed in a routine setting of Lyon’s Hospital as described in the kit inserts of the corresponding commercial assay used (Siemens, Germany).

### Affinity-purified antibodies and immunofluorescence

50 ml from a 51 years old man TcCRA-positive plasma sample were used for affinity-purification. Gammaglobulins fraction was precipitated with 40% Ammonium Sulfate. Pellet was centrifuged at 4.000 g then dissolved in 30 ml of PBS after discarding the supernatant. When fully dispersed, the solution was dialyzed against PBS at 4°C overnight with two buffer changes. The dissolved fraction was used for immunoaffinity purification as follows: 20 mg Dynabeads MyOne™ Streptavidin T1 (Invitrogen 656.01) were incubated for 1 hour with 20 µg/ml of a biotinylated TCSP; the beads were then washed 3 times with PBS-Tween 20 and incubated with PBS 1% BSA for another hour to saturate all the unspecific binding sites. Washed one time after the saturation, the beads were incubated with 10 ml of the precipitate (Total IgGs) for one hour. After incubation the beads were washed 3 times with PBS-Tween 20, and incubated for 10 min with a glycine-HCL (pH 2.5) to elute TcCRA. After elution, pH was immediately neutralized to pH 7.5 by adding NaOH (1N). All incubations were performed at room temperature and before each step a magnet was used for 2 min to isolate beads from supernatant.

After the immunopurification, TcCRA solution (300 µg/mL) was diluted 1/50 then incubated in a wet chamber on an immunofluorescence assay used in routine screening (epimastigotes glass slides;from Biomérieux/IMUNOCRUZI) during 1 h at 37°C. Later, the slide was washed three times in PBS-Tween and incubated at dark with a diluted ready to use FITC-conjugated anti-Human IgG (NOVA Lite kits, INOVA Diagnostics) during 1 h at 37°C, then washed three times in PBS and mounted in buffered glycerin for observation under a fluorescence microscope (excitation at 488 nm) 60x. A negative control was obtained by using an irrelevant purified human IgG negative to TCSP antigen under identical concentration and testing conditions.

### Statistical analysis

Descriptive statistics are presented as frequencies (percentages) for categorical variables and as means (SD) for continuous variables. The Mann Whitney U test was used to compare age between sexes and Fisher’s Exact test was used to test equality of the proportion of males and females in the TcCRA positive group (Table 2). To investigate the equality of viral serology of other viruses and TcCRA status, the null-hypothesis that the proportion of discordant test results equals zero is tested using a single proportion test (Table 3). All statistical analyses were performed using SPSS software (SPSS version 17.0).

**Table 2:** TcCRA seroprevalence in blood donors. Gender and age are compared.

Blood donors (n = 210)	Male (n = 134)	Female (n = 76)	*P values*
TcCRA positive (%)	57 (42.5)	38 (50)	0.315
Age, mean years (SD)	41.1 (14.6)	36.7 (14.4)	0.037

**Table 3:** Different viral serologies as compared to TcCRA.

Viral serologies (IgG measurements)		TcCRA Negative	TcCRA Positive	Disagreements (%)
Adenovirus	−	3	1	20/52
	+	19	29	(38.4)
				
Epstein Barr Virus (EBV)	−	15	16	129/257
	+	113	113	(50.1)
				
Enteroviruses (Echo-Coxsackie viruses)	−	15	18	48/100
	+	30	37	(48)
				
Human Herpes Virus 6 (HHV6)	−	11	6	53/110
	+	47	46	(48.1)
				
Mumps	−	7	3	39/82
	+	36	36	(47.5)
				
Parvovirus	−	16	16	56/110
	+	40	38	(50.9)
				
Measles	−	16	15	53/104
	+	38	35	(50.9)
				
Respiratory Syncytial Virus (RSV)	−	2	1	26/44
	+	25	16	(59)
				
Rubella virus	−	4	1	45/87
	+	44	38	(51.7)
				
Varicella Zoster Virus (VZV)	−	3	2	95/185
	+	93	87	(50.2)
				
Herpes Simplex Viruses 1&2 (HSV)	−	30	19	89/191
	+	70	72	(46.5)
				
Cytomegalovirus (CMV)	−	53	49	132/263
	+	83	78	(50.1)

*P* values calculated by using single proportion tests are <0.0001 for each viral serology.

## Results

### Immunofluorescence

**Figure 1:**
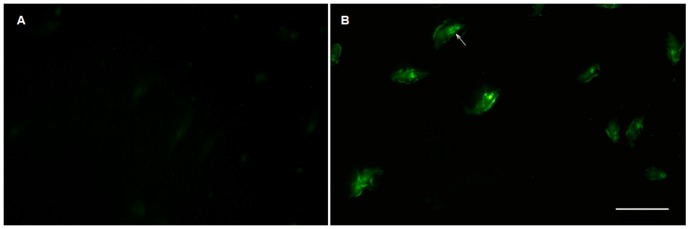
Immunoreactivity of affinity-purified TcCRA (B) as compared to a negative control that uses irrelevant human antibody (A) on *Trypanosoma cruzi* epimastigotes as shown by fluorescence microscopy. The horizontal bar embedded in the image B represents a 20 µm scale.

Affinity-purified TcCRA were tested by immunofluorescence on *T. cruzi* epimastigotes slides. These purified antibodies reacted positive with the fixed parasite giving strong fluorescence signals (Figure 1) as compared to the negative control antibodies.

### Serological testing

#### Sera from adult blood donors and pediatric patients

**Figure 2:**
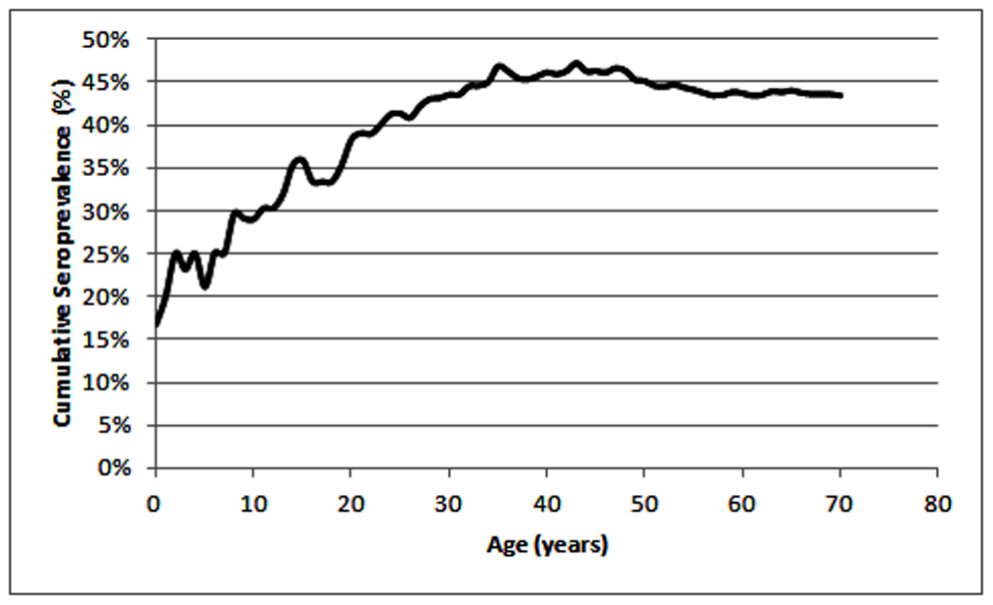
Cumulative seroprevalence of TcCRA in function of age (1 year intervals).

We tested TcCRA in sera obtained from blood donors qualified after negative screening for all mandatory infectious markers (n = 210). Since seroprevalence in healthy young individuals (below 18 years old), for ethical reasons, is relatively complex to evaluate, we estimated the seroprevalence in pediatric patients. Sixty-nine serum samples from hospitalized children were tested to evaluate the seroprevalence of TcCRA in a lower age range than for blood donors. By merging the two groups, Figure 2 shows a steady increase of TcCRA seroprevalence from childhood to adulthood. There was no significant difference related to gender in the adult blood donors group (Table 2).

#### Sera from patients tested for different viral serologies

395 serum samples obtained from patients referred for viral serology testing by different hospital wards in Lyon were tested for the presence of TCcRA. Each sample was analyzed in a clinical laboratory for at least one of the IgG antibodies specific to viral serologies. Table 3 shows the seroprevalence of TcCRA in each category of negative and positive viral serology. In this table, we tested TcCRA status against presence/absence of different viral serologies and p<0.0001 was obtained, providing evidence for the difference between TcCRA and other viral serology markers.

#### Sera of *T. cruzi* infected patients

A cohort of 79 patients infected by *T. cruzi* and collected in Brazil for a study on Chagas disease was used in an ancillary study to evaluate the specific antibodies to the ribosomal protein antigen TCSP [22]. 96% (76/79) of samples were found reactive to this antigen. Under the testing conditions of such samples, no discrimination was feasible between genuine anti-*T. cruzi* antibodies and the cross-reactive TcCRA.

### Sequence Homologies

**Figure 3:**
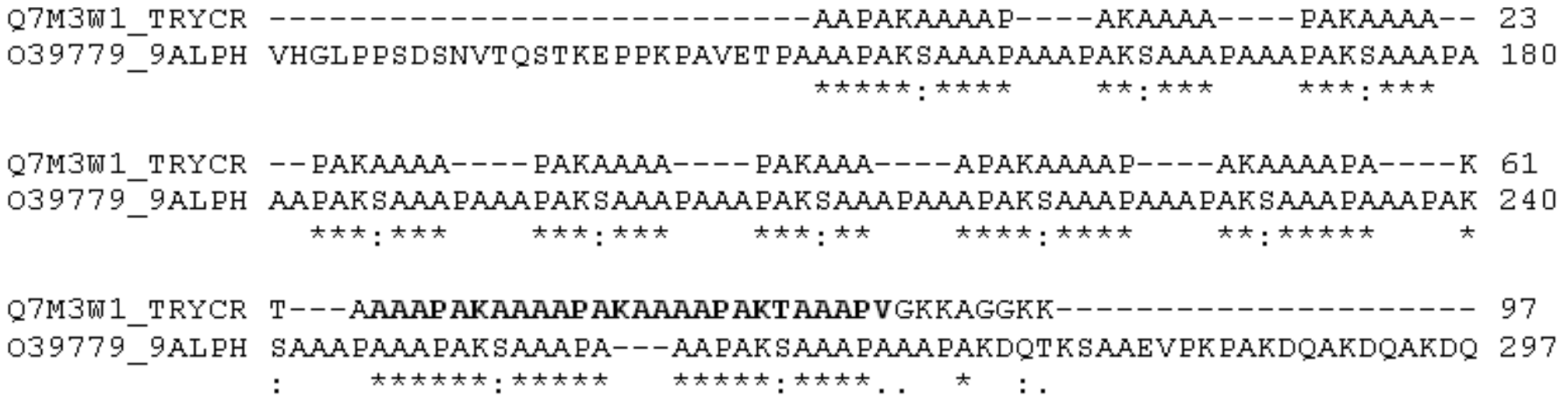
Alignment between the repetitive fragment Q73MW1 and the tegument protein of equine herpes virus.

A BLAST search with the TCSP sequence did not reveal any similarity with classical and frequent infectious agents that could explain the observed seroprevalence levels. We also performed searches with the Q7M3W1 repetitive fragment containing TCSP. Q7M3W1 fragment has been classified in the superfamily varicella-zoster virus gene 22. Searches indeed revealed a significant similarity with a tegument protein that belongs to a herpes virus family member [O39779, equine herpes virus 1, Fragment, ORF24, identity = 57%, score = 248, E-Value =  1.0E-19] (Figure 3). No other similarity with members of the Herpes family (and especially human) was evidenced through this search.

We then checked if the *in silico* similarity between TCSP and the equine herpes virus had a biological value. The equine peptide was synthesized and tested with TcCRA seropositive samples. Although linear sequence identity of the tested antigens exceeds 69%, only a minor fraction (below 1%) of samples reacted to the herpes equine antigen and no correlation was identified between both serologies (data not shown).

## Discussion

We have discovered novel antibodies, called TcCRA, observed in around 45% of French blood donors though detected with an antigen derived from a Latin American parasite.

French individuals are virtually not exposed to *T. cruzi*. We however observed at a high frequency binding reactions in the ELISA tests performed on French blood samples. This strong TcCRA cross-reactivity with *T. cruzi* was further evidenced by the specific reaction obtained between affinity-purified TcCRA and *T. Cruzi* epimastigotes in immunofluorescence assays used for routine serodiagnosis. This led us to search for characteristics of the trigger immunogen through different approaches.

We estimated seroprevalence in different groups of individuals. Our data show a steady increase of seroprevalence from early childhood to an approximate level of 47%. No differences were found between men and women. This suggests a widely spread immunogen, acquired in childhood, probably latent after acquisition or initiating an autoimmune process, which would explain its immunity maintenance during adulthood.

In a second approach, we investigated potential homologies with other antigens than the one from *T. cruzi*. We could not identify any significant similarity to known pathogens with such a high level of prevalence through our BLAST searches. Intriguingly enough, the annotation of Q7M3W1 (eukaryotic) indicated a clustering in the “super family” of varicella-zoster virus gene 22 protein, through its homology with the equine ORF24 genes. So far there is no history of Equine Herpesvirus transmission to human [23] and our in-vitro experiments revealed no interaction between TcCRA and the homologous peptide derived from ORF24.

Without any conclusive outcome to the in-silico search of potential homologies, we explored the path of in-vitro cross-reactivity with the commonly tested viral agents widely prevalent within the French territory. We could not find any direct association, in the sera we tested, between TcCRA and any of the analyzed viral serologies, whether they belong to the Herpes virus family (namely HSV1/2, HHV6, EBV, CMV and VZV), or other viruses (Adenovirus, Parvovirus B19, Mumps virus, Rubella virus, Respiratory Syncytial Virus, Measles and Enterovirus).

Based on our observations so far, we thus propose that the trigger immunogen, still to be characterized, could be a variant of a known infectious agent or even a new one in view of the lack of correlation with widely spread viruses. It could be of parasitic nature: cross-reactivity between *T. Cruzi* and closely related species like *T. rangeli* of *Leishmania* or with more distant species has indeed been observed [14]. However, though some of these species are found in Europe, their presence has not been reported in France. They are thus unlikely to explain the elevated seroprevalence levels of TcCRA.

Overall, the seroprevalence rate and age of acquisition hint at a cosmopolitan distribution for the target immunogen, which is likely to be present in the endemic, as well as in non-endemic, zones of *T. cruzi*. Given the strong cross-reactivity of TcCRA with the Latin American parasite, these antibodies could explain a certain amount of false reactivities typically observed in *T. cruzi*-screening assays that use whole extracts of the parasite. This hypothesis is reinforced by our immunoassay results on *T. Cruzi* infected patients (96% of positive reactions in a group of 79 individuals) showing that TCSP is an immunodominant antigen for Tc immunoassays and concomitantly can yield false reactivities in individuals uninfected by Tc. To resolve such false reactivities and achieve high assay specificity, Oelemann et al. made a careful selection of protein composition that excluded some of the *T. cruzi* antigens (such as TCSP) that might sustain the cross-reactivities [16].

We are confronted here to a case of cross-reactivity between distinct infectious agents striking by its extent: elevated prevalence estimated in different groups of individuals, either healthy (blood donors) or not (patients with diverse pathologies). Other examples in humans have been described in the literature but not necessarily on humoral immunity, such as cross-reactivity between HCV and Influenza virus, impacting cellular immunity. Wedemeyer et al. were indeed able to expand T-cells specific to a peptide derived from NS3 protein of HCV from blood of 9 of 15 HCV-negative blood donors (60%) [24]. They further confirmed that the cross-reactivity originated from a peptide of Influenza virus. They concluded on the influence past exposure to pathogens might have on host cellular responses to a new infection. Could there be a similar mechanism in Latin American patients having first acquired TcCRA target immunogen, where the immune response takes a different path in the course of an infection by *T. cruzi* due to the presence of cross-reactive antibodies?

Most of documented cross-reactivity cases refer to an infectious agent and a self-antigen rather than between pathogens. *T. cruzi* is an exemplar of molecular mimicry between some of the epitopes it bears and human proteins, which may initiate autoimmune disorders and alter several organs. This has been in particular proposed to explain (at least partially) Chagas pathogenesis through the induction of autoantibodies against Beta1-adrenoreceptors, provoking dilated cardiomyopathies (DCMs) [5]. Based on the reported presence of autoantibodies against the Beta1-adrenoreceptor in all forms of DCMs (idiopathic and chagasic), Levin and Hoebeke have proposed a parallel between both forms and a consensus peptide common to *T. cruzi*, to the Beta1-adrenoreceptor and to other pathogens that could explain the origin of idiopathic forms [25]. Their research works lead us to raise the question of potential analogous autoimmune mechanisms that may be elicited by the peptide sequence shared by *T. cruzi* and TcCRA target immunogen. This may be one possible explanation for the persistence of TcCRA during adulthood, another one being a repetitive exposure to the antigen through a latent infection. Exploring this hypothesis will be pursued, with the full awareness of the difficulty of the task. First, the long time span (several years or even decades) between the autoantibodies generation and the development of the associated clinical disease indeed requires long-term epidemiologic studies, taking many years to produce results [26]. Moreover, the occurrence of autoimmune diseases is suspected to be associated with a confluence of several factors such as genetic predisposition and environmental exposure [3]. Another source of complication in comprehending autoimmune processes is due to the low prognosis value of functional autoantibodies. Indeed only low correlations are found between autoantibodies and a given disease severity [27]. This extensive complexity probably explains why direct evidence about infections and their contribution to autoimmunity have been established only in a few instances, such as Guillain-Barré syndrome [28] and rheumatic fever [29].As for Chagas disease, some controversies are still under debate about its origin: either autoimmunity or parasite persistence.

## Conclusion

The present report unveils a possible antigen mimicry characterized by a serological reactivity to a well-defined *T. cruzi* antigen in blood samples from individuals not exposed to the parasite. The measured seroprevalence of such cross-reactivity is in favor of a highly prevalent immunogen, acquired in childhood, which doesn’t seem to be associated with common known pathogens in clinical routine. Additional studies are required to identify the candidate agent probably bearing a structural immunogenic motif similar to the ribosomal antigen of *T. cruzi*. This initial work will serve as the basis for organizing prospective clinical investigations, where we will pursue the analysis of TcCRA in different groups of individuals (diseased and healthy) with the aim to identify its potential clinical significance and etiology.
